# cAMP Biosensors Based on Genetically Encoded Fluorescent/Luminescent Proteins

**DOI:** 10.3390/bios11020039

**Published:** 2021-01-31

**Authors:** Namdoo Kim, Seunghan Shin, Se Won Bae

**Affiliations:** 1 Department of Chemistry, Kongju National University, Gongju 32588, Korea; ndkim123@kongju.ac.kr; 2 Green Chemistry & Materials Group, Korea Institute of Industrial Technology (KITECH), Cheonan 31056, Korea; shshin@kitech.re.kr; 3 Department of Chemistry and Cosmetics, Jeju National University, Jeju 63243, Korea

**Keywords:** cAMP, fluorescent protein, luciferase, circular permutation, FRET, BRET, non-invasive imaging, genetically-encoded biosensors, Epac, PKA

## Abstract

Cyclic adenosine monophosphate (cAMP) plays a key role in signal transduction pathways as a second messenger. Studies on the cAMP dynamics provided useful scientific insights for drug development and treatment of cAMP-related diseases such as some cancers and prefrontal cortex disorders. For example, modulation of cAMP-mediated intracellular signaling pathways by anti-tumor drugs could reduce tumor growth. However, most early stage tools used for measuring the cAMP level in living organisms require cell disruption, which is not appropriate for live cell imaging or animal imaging. Thus, in the last decades, tools were developed for real-time monitoring of cAMP distribution or signaling dynamics in a non-invasive manner. Genetically-encoded sensors based on fluorescent proteins and luciferases could be powerful tools to overcome these drawbacks. In this review, we discuss the recent genetically-encoded cAMP sensors advances, based on single fluorescent protein (FP), Föster resonance energy transfer (FRET), single luciferase, and bioluminescence resonance energy transfer (BRET) for real-time non-invasive imaging.

## 1. Introduction

3′-5′-cyclic adenosine monophosphate (cAMP) is an important second messenger in biological processes, especially in signal transduction, in almost all organisms. Adenylyl cyclase (AC) converts adenosine triphosphate (ATP) into cAMP, which requires the cleavage of PPi (pyrophosphate). In prokaryotic cells, the cAMP level varies with the used carbon source, such as glucose. Lac operon [1], for example, is positively regulated by cAMP in glucose-deficient environments through the binding to the cAMP receptor protein (CRP), which engages with the Lac operon. In eukaryotic cells, the major role of cAMP is to activate protein kinase A (PKA). PKA is an inactive holoenzyme with two regulatory subunits and two catalytic subunits. Upon the binding of cAMP, the catalytic subunits dissociate from the regulatory subunits and are able to phosphorylate other proteins or targets that need to be phosphorylated. Phosphorylated proteins can be activated or deactivated according to their physiological roles [2]. Besides PKA, cyclic nucleotide regulated ion channels (cyclic nucleotide-gated (CNG) and hyperpolarization-activated cyclic nucleotide-gated (HCN)) [3] exchange proteins that are directly activated by cAMP (Epac1 and Epac2) [1,2,4,5], Popeye domain containing (POPDC) proteins [6], and cyclic nucleotide receptor involved in sperm function (CRIS) [7] are known cAMP effector proteins. Some ion channels can be directly activated by phosphorylated proteins. Target G protein-coupled receptors (GPCRs) are pharmacologically important and crucial for cAMP activation and further signal processing [8].

Several tools and devices have been developed to investigate the cAMP levels in living cells or organisms. However, these yield a signal with a poor spatial and temporal resolution. cAMP measurements from lysed cells only allow us to measure the cAMP level at single-time points [9]. Thus, it was impossible to make a graph regarding the [cAMP] fluctuation on a single cell with time. Additionally, non-invasive imaging was not easy to achieve since the injection of artificial sensors might affect the physiology of living species by disrupting their normal cellular function.

Purified regulatory subunits (R) and catalytic subunits (C) of cAMP-dependent protein kinase were labeled with tetramethylrhodamine isothiocyanate and fluorescein isothiocyanate, respectively, for Föster resonance energy transfer (FRET) measurement induced by cAMP binding/unbinding. This sensor was injected into BC3H1 and REF-52 cell lines and the emission ratio changes were recorded or imaged [10]. The same sensor was used to study cAMP dynamics in *Aplysia* sensory neurons [11]. 

However, the need to inject sensor components limited application of this sensor in living cells and animals imaging. Thus, genetically-encoded cAMP sensors that do not need to be injected have been developed for non-invasive imaging. Here, we described several types of genetically-encoded cAMP sensors and their applications based on their luminescent units, cAMP biding domain, and tested species.

## 2. Fluorescence-Based cAMP Sensors

### 2.1. Single Fluorescent Protein (FP)-Based cAMP Sensors 

The simplest system of genetically-encoded cAMP indicators might be the one using single fluorescent proteins. Usually, fluorescent proteins are engineered to undergo fluorescence intensity changes upon binding/unbinding of cAMP. For this purpose, the cAMP-binding domain is fused with an FP. These sensors have advantages over FRET-based sensors: (1) They only need one emission wavelength for imaging and (2) they can be used for multi-color imaging, enabling the use of images of living cells with two- or three-colors, without any spectral overlap.

#### 2.1.1. cAMP Difference Detector in Situ (cADDis) 

Circularly permuted green fluorescent protein (cpGFP) was inserted between the catalytic and the enzymatic domains of a guanine nucleotide exchange factor Epac2 (hinge region) [12,13]. Epac2 undergoes a conformational change upon the binding/unbinding of the cAMP molecule. Extensive studies on the position of cpGFP and the linker composition between cpGFP and Epac2 produced two oppositely working sensors whose fluorescence signal increased/decreased in the presence of cAMP (upward and downward, respectively). The sensor whose fluorescence signal decreased showed a better dynamic range (~35% of fluorescence decrease) and was named the cADDis. For the in vitro test as a proof-of-concept, cADDis protein was purified and the cAMP concentration-dependent fluorescence was tested using a plate reader. cADDis has a similar sensitivity to that of the other Epac-based sensors. However, its *K_d_* is relatively high in vitro (10~100 μM). When the construct was transfected into baculovirus, a mono-exponential increase of the total fluorescence was observed. Finally, HEK293T cells were transfected and the EC_50_ for D_1_ dopamine receptor, endogenous adenosine receptor, and endogenous β–adrenergic receptor were measured (2.9, 426, and 3.5 nM, respectively). 

Heterodimeric G protein alpha subunit I (Gαi) could inhibit Gαs-stimulated AC, which leads to a decrease of the cAMP concentration. According to the FPs used, a green (cpmNeonGreen) and red (cpmApple) version of cADDis could be generated [14]. HEK293T cells expressing active Gαs showed a decreased cADDis signal, indicative of an elevated cAMP level. In contrast, Gi with several receptor agonists showed an increased cADDis signal. Furthermore, cADDis was able to discriminate the signals mediated by Gαs or Gαi. Finally, a green cADDis was co-transfected with R-GECO, a red fluorescent genetically-encoded Ca^2+^ indicator for optical imaging, and with the D2 receptor into HEK293T cells to study the regulation of AC by Ca^2+^. The cAMP level increased when β2-adrenergic receptor (AR) was activated by isoproterenol (adenylyl cyclase activity was risen without any effect on calcium ion). The addition of quinpirole suddenly increased the intracellular Ca^2+^ level since it activates the D2 receptor. In summary, direct and live cell assay of cAMP level change by Gαi and Gαs signaling was enabled with this sensor.

When fibroblasts are consistently in the activated form, harmful fibrous materials are deposited in the lungs, causing pulmonary fibrosis [15]. It has been known that cAMP prevents the fibrotic process, and that prostaglandin E_2_ (PGE_2_) could stimulate cAMP production. Human fetal lung (HFL)-1 cells were used to investigate the long-term exposure of lung fibroblasts to PGE2 and other molecules that stimulate cAMP production [16]. The cAMP level was monitored using cADDis. At a PGE_2_ concentration of 100 pM, there was little change in fluorescence. However, the fluorescence changes became significant as PGE_2_ concentration increased from 10 nM to 1 μM. Interestingly, the pre-treatment of isoproterenol or forskolin did not affect the fluorescence signal. By contrast, prostaglandin E (EP)_2_ receptor (EP_2_R) agonist desensitized the PGE_2_ response, while EP_4_R agonist did not. Finally, the PGE_2_ response was desensitized upon enhancement of phosphodiesterase (PDE) activity. 

Red cADDis (R-cADDis) was transfected into HEK293T cells with blue photo-activated cyclase (bPAC), which is activated using 480 nm light to convert ATP into cAMP [17]. Upon blue light illumination, R-cADDis fluorescence increased, meaning that bPAC was activated by the blue light. The red fluorescence of the control, which did not contain bPAC, did not change. This increase became saturated when the power of the blue light reached ~35 mW/cm^2^, which is ~50% of the output power of the LED.

#### 2.1.2. GAkdYmut 

GFP has 11 β-strands that form a barrel-shaped structure. The amino acid sequence Ser-Tyr-Gly (SYG), from the sixty-fifth to the sixty-seventh residue, undergoes an autocatalytic reaction to become a chromophore. Mutations in the seventh β-strand make the chromophore sensitive to conformational changes due to the interaction of GFP with the other domains. Originally, the FRET-based PKA sensor AKAR2 was modified so that the resulting fusion proteins had both GFP and quencher which was suitable for single-wavelength sensors. However, Agnès Bonnot et al. found that a mutant GFP alone (without quencher) was enough to be used as a sensor [18]. The PKA-sensing domain was conjugated to a mutant GFP, and the conformational changes affected the fluorescence lifetime and intensity. In a baby hamster kidney (BHK) cell experiment, forskolin induced fluorescence change significantly (~60%). This single-wavelength PKA sensor was in neurons of brain slices to report PKA activity. After viral transfection, reversible PKA activity was observed upon activator molecules. Finally, neuronal processes were imaged with 2-photon microscopy to monitor PKA dynamics. Fluorescence change in the dendritic tree was monitored with high-resolution.

#### 2.1.3. Fluorescent cAMP Indicator (Flamindo) 

To study the interplay between cAMP and Ca^2+^ in exocytosis, Flamindo, using a yellow fluorescent protein (YFP), was developed. Epac1 (exchange protein directly activated by cAMP 1) was inserted into Citrine [19], a YFP, and cAMP binding induced conformational changes in the vicinity of the YFP chromophore [20]. This led to changes in YFP fluorescence intensity about ~50%. COS-7 cells expressing Flamindo showed a fluorescence decrease in the presence of cAMP. Furthermore, the cAMP concentration was elevated upon glucose stimulation in MIN6 cells due to the activation of adenylate cyclase 1 (ADCY 1).

#### 2.1.4. Flamindo2 

Even though Flamindo has previously been successfully applied to mammalian cells as a cAMP sensor in the cytoplasm and plasma membrane, it is not bright enough and has a small dynamic range. Thus, an improved version, Flamindo2, was developed by optimizing the linker sequence of each terminal of the cAMP-binding domain near the chromophore of Flamindo [21]. In Flamindo2, the linker at position 144 consists of LRGALKK, whereas Flamindo has an LRG at the same position. A saturating concentration of cAMP (1 mM) induced a 75% decrease in the fluorescence intensity (4-fold change), which is a two-fold improvement compared to Flamindo (50% decrease (2-fold change)). Furthermore, an 8-fold brightness enhancement was achieved. In live-cell imaging, COS-7 cells transfected with the Flamindo2 construct showed a 70% decrease in fluorescence intensity upon forskolin application. The fluorescence intensity of 3-isobutyl-1-methylxanthine (IBMX) and 8-Br cAMP also decreased by 40~55% and 70%, respectively. Finally, two-color imaging of cAMP and Ca^2+^ in HeLa cells was conducted using Flamindo2 and R-GECO [22]. R-GECO signal transiently increased, and Flamindo2 signal was gradually decreased, followed by recovery in the presence of 100 μM of noradrenaline, which is consistent with previous studies. 

#### 2.1.5. Pink Flamindo 

**Figure 1:**
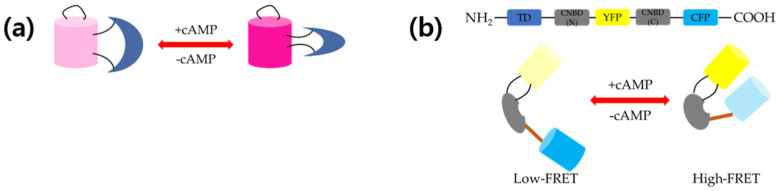
Schematic representation of (**a**) structure and working mechanism of Pink Flamindo and (**b**) protein kinase A (PKA)-based 3′-5′-cyclic adenosine monophosphate (cAMP) sensor cAMP universal tag for imaging experiments (CUTie). Pink Flamindo is a single fluorescent protein (FP)-based sensor. Binding of cAMP enhances fluorescence and *vice versa*. Without cAMP, two fluorescent proteins are apart from each other (low Föster resonance energy transfer (FRET)), whereas two FPs are close to each other in the presence of cAMP (high FRET).

Since the previously developed single FP-based cAMP sensors used GFP or YFP, an expansion of the available color sets was needed to combine them with other blue light-excitable sensors. Thus, a new red FP-based cAMP sensor was developed. To improve the dynamic range of the sensor, Shogo Matsuda et al. used the same strategy as that used for cGMP sensor development [23]. The residues from the two hundred and fifth to the three hundred and fifty-third position of mEpac1 were incorporated into the one hundred and fiftieth position of mApple; then, the two linkers between mApple and mEpac1 were optimized [24]. Compared to Flamindo or Flamindo2, this sensor had a red-shifted emission (λ_ex_: 567 and λ_em_: 590 nm). A 4.2-fold increase in fluorescence was observed in the presence of 100 μM cAMP. Figure 1a shows fluorescence changes upon binding of cAMP to Pink Flamindo. In addition, the response time was quite low (within 5 s), even at low cAMP concentrations. However, the incubation temperature of the transfected cells should be low to accelerate chromophore maturation for a better brightness. The *K_d_* for cAMP was 7.2 and it was 94 μM for cGMP, which were slightly higher values than those of Flamindo2. In live-cell imaging, HeLa cells expressing Pink Flamindo showed an increased fluorescence intensity upon treatment with 100 forskolin and 200 μM IBMX, and a decreased fluorescence intensity in the presence of 100 μM 2′,5′-dideoxyadenosine (DDA). Co-transfection of Pink Flamindo with G-GECO into MIN6-m9 cells enabled the dual-color imaging of cAMP and Ca^2+^, simultaneously. Finally, cerebral cortical astrocytes of mice were imaged using an in vivo two-photon microscope. The pink Flamindo gene was delivered by using Adeno-associated virus (AAV) with human Glial fibrillary acidic protein (GFAP) promoter. The application of 50 μM forskolin and 500 μM IBMX increased the fluorescence intensity in astrocytes.

#### 2.1.6. Red Fluorescent Indicator for cAMP (R-FlincA) 

To overcome the low ligand affinity and the poor dynamic range of the existing fluorescent cAMP sensors, circularly permuted red fluorescent protein, mApple (cp146mApple) [25,26], was inserted into several positions of the cAMP-binding motif of PKA regulatory subunit Iα [27]. This new sensor was named R-FlincA. Several insertion positions were studied and the most prominent one exhibited an 860% fluorescence intensity change at physiological pH and a high affinity (*K_d_* = 0.3 μM) for cAMP. Together with B-GECO1 and ATeam1.03, which are the biosensors of Ca^2+^ and ATP, respectively, three-channel, four-color imaging was done in pancreatic β-cell line MIN6, upon glucose uptake. In addition, intra/extra cellular cAMP was imaged simultaneously using R-FlincA and PKA Riα #7 (CFP-YFP FRET) with *Dictyostelium discoideum* cells. Intracellular cAMP concentration oscillated similarly with extracellular cAMP concentration.

#### 2.1.7. cAMPr 

Epac-based cAMP sensors usually show faster kinetics and a larger dynamic range, compared to PKA-based ones. However, cADDis has a high *K_d_* in vitro; thus, it might not be suitable for animal imaging. A PKA-based cAMP sensor was developed to find bright constructs in bacteria; then, embryonic stem (ES) cells were transfected with the selected constructs. The catalytic subunit and the regulatory subunit of PKA (PKA-C and PKA-R, respectively) were fused to both terminals of cpGFP, resulting in two constructs (C-G-R and R-G-C, respectively) [28]. Only the C-G-R constructs showed a response to forskolin in ES cells. Hackley et al. selected the brightest bacteria colonies using flow cytometry, and then moved to a eukaryotic system to test the cAMP response. ES cells were transfected with constructs and the forskolin response was further tested, undergoing a 30~40% change in fluorescence. After sequencing, it was revealed that the N-terminal linker was PG and that of the C-terminal was TAPPAC in four constructs out of six fluorescent ones. The other two constructs have original PG and AC linkers. The construct with the longest C-terminal linker showed faster kinetics against forskolin than shorter ones. This variant was named as cAMPr. Even though cAMPr has a lower dynamic range than cADDis, cAMPr showed a linear response to forskolin, while cADDis showed a sigmoidal response. cAMPr was tested in both mammalian neurons and *Drosophila* pacemaker neurons. Finally, cAMP and Ca^2+^ were imaged simultaneously in whole brains, using cAMPr and RCaMP1h. The fluorescence increase of RCaMP1h occurred first in projections, then cell bodies underwent this fluorescence change [29]. In both projections and cell bodies, RCaMP1h showed a fluorescence change before cAMPr did.

### 2.2. FRET-Based cAMP Sensors 

#### 2.2.1. PKA-Based Sensors 

Holoenzyme PKA is the main effector of cAMP, which consists of two regulatory subunits (RI and RII) and two catalytic subunits (C) [30,31]. cAMP activates PKA, leading to the dissociation of the catalytic subunits from the regulatory subunits; then, the free catalytic subunits can phosphorylate their substrates. S65T mutant GFP was fused to the RI or RII subunit and enhanced blue fluorescent protein (EBFP) was fused with C [32]. Several possible combinations were tested, and it was found that the RII-EBFP/C-GFP S65T pair showed the best performance in CHO cells. As expected, the FRET ratio decreased with time, after the addition of cAMP and forskolin. This sensor was also used to study the compartmentalization of PDE, an enzyme responsible for degrading cAMP in live neonatal rat cardiomyocytes [33]. PDE3 and PDE4 have been known to be the major enzymes that control the cAMP level in rat ventriculocytes. In their study, PDE4 seemed to be more responsible for the cAMP level than β–agonists stimulation. The different roles of PDE3 and PDE4 might originate from their localization in different microdomains.

The CFP-RIIβ and YFP-C FRET pair was used to investigate cAMP dynamics in live *Dictyostelium discoideum* cells [34]. Western blot assays revealed that the expression levels of each construct were different; in particular, the expression level of the donor was too low compared to that of the acceptor. Furthermore, the development of the cells was somewhat defective when expressing this sensor. Thus, the tetrameric sensor couldn’t be able to be used in this study. Thus, monomeric ECFP-CNBD from the RIIβ-EFYP construct was tested. The addition of a chemoattractant (10 μM) to cells induced a decreased FRET efficiency (by 20.4%). In addition, it was revealed that this increased cAMP level was due to adenylate cyclase A (ACA), but not to adenylate cyclase B (ACB). Next, RegA phosphodiesterase was responsible for the degradation of intracellular cAMP, while *regA* cells showed a much smaller FRET ratio change. Finally, *regA* cells were able to respond to the cAMP released from the micropipette by moving toward the micropipette tip.

The R subunits of PKA were linked to CFP and the C subunits were linked to YFP to study the cAMP localization in rat cardiomyocytes [35]. Rat ventricular cardiomyocytes co-expressing both constructs emitted a strong fluorescence in the cytosol and in thin parallel striations. The addition of IBMX caused the complete dissociation of R-CFP and C-YFP, and this effect was reversible. FRET kinetics was recorded to study the cAMP fluctuations by adding norepinephrine (NE) or NE/IBMX to neonatal cardiomyocytes. It was also found that stimulation by NE could induce localized cAMP level fluctuations.

cAMP signaling is important in cardiac contractility, and tools for the precise measurement of compartmentalized cAMP at certain locations were needed. When CHO cells were transfected with Epac1-camp sensor and treated with a saturation dose of forskolin and IBMX to ensure a maximal response at any site, the dynamic ranges of the sensor were not consistent [36]. In the case of PDE4A1-Epac1-camp, even no response was observed. cAMP universal tag for imaging experiments (CUTie), a new FRET-based cAMP sensor, used Epac1-camp to make a universal cAMP sensor [37]. YFP was incorporated into the middle of the cyclic nucleotide-binding domain (CNBD) of PKA RIIβ and CFP was conjugated to the C-terminal of CNBD. Figure 1b shows how cAMP binding/unbinding affected the FRET signal of CUTie. This novel sensor showed almost the same dynamic ranges for all target domains tested in CHO cells. Neonatal rat ventricular myocytes (NRVM) expressing three different target-specific CUTies showed the expected localization results (AKAP18δ/SERCA/PLB: Localized at the sarcoplasmic reticulum (SR); TPNI/TPNT/TPNC: Localized at the myofilament; and AKAP79/β-AR/adenylyl cyclase/LTCC: Localized at the plasma membrane).

CUTie that targets A-kinase anchoring protein 79 (AKAP79) was transfected into rat ventricular myocytes [38]. Both AKAP79-CUTie and AKAP79_mut_-CUTie sensors were localized in the membrane. However, AKAP79_mut_-CUTie did not bind to the RIIα subunit of PKA due to the mutations. The anchoring of both sensors did not affect the local cAMP dynamics. AKAP79 and AKAP79_mut_ were fused to the PKA activity reporter AKAR4. Neuronal rat ventricular myocytes expressing both sensors showed membrane-localized signals due to binding of AKAP79 with phosphatidylinositol-4,5-bisphosphate (PIP2) [39]. Similar FRET ratio changes were observed upon using 0.05 nM isoproterenol. It was shown that anchoring of PKA to AKAP79 did not affect local cAMP signal dynamics using 0.5 nM of isoproterenol.

Although FRET has been used in a wide range of biological studies, some FRET pairs have a small dynamic range due to the distance and orientation sensitivity of fluorophores. Thus, optimizing several parameters to increase the dynamic range of the FRET pairs has received much attention. All possible combinations of the wild-type and cp173 version of CFP and YFP, as well as their relative order in the sensor, were tested to find the ones with the best dynamic range [40]. These sensors were fused to Ca^2+^ sensor, cGMP sensor, and cAMP sensor. The best cAMP sensor was the one with the cAMP-binding domain of PKA RIα sandwiched with cp173Venus and ECFP at the N- and C-terminal, respectively. It showed more than 20% FRET signal change in lysed HEK293T cells, while no other sensors showed meaningful changes. When this sensor was purified, it exhibited a 38% change in the dynamic range. In the extracellular experiment with *Dictyostelium discoideum* cells, this sensor monitored the FRET change periodically, in 7-min intervals.

#### 2.2.2. Epac-Based Sensors 

PKA-based sensors have a few drawbacks: (1) Slow response time (2), small dynamic range (3), need to transfect two separate constructs (4), low S/N ratio, and (5) problems due to multi-subunits. Thus, their application should be limited. Epac-based cAMP sensors have better characteristics than PKA-based sensors to monitor cAMP dynamics in vivo. Epac1 was sandwiched between CFP (N-terminally) and YFP (C-terminally) to measure the FRET ratio upon binding/unbinding of cAMP [41]. Human A431 cells expressing the CFP-Epac-YFP sensor increased their CFP fluorescence upon addition of forskolin, implying an increased cAMP level. Similar results were obtained with HEK293, N1E-115, and MCF-7 cells. Thus, binding of cAMP induced conformational change in Epac1. Kinetic analysis revealed that the FRET ratio began to decrease within a few seconds after the addition of forskolin and reached a minimum level in 2~3 min.

When existing FP-based sensors were transfected into excitable cells, such as neurons, the pH slightly changed, which greatly affected the brightness of FP. To address this problem, a less pH-sensitive cAMP sensor was developed [42,43,44]. Since the normally used ECFP and EYFP are pH sensitive, using them in neuronal cells must produce an artifact. Cerulean [45] and citrine [46] were fused to the adenylyl cyclase 8 (AC8) to investigate the regulation of the AC8 in MIN6 cells [47]. First, the citrine/cerulean (Ci/Ce) Epac2-camp (type 2 exchange protein activated by cAMP) sensor was transfected into HEK293 cells and lysed to measure the FRET in vitro. YFP/CFP Epac2-camp and Ci/Ce Epac2-camp had a similar EC_50_ for cAMP (450 and 545 nM, respectively). Ci/Ce Epac2-camp fluorescence was less pH sensitive than that of the YFP/CFP Epac2-camp, upon addition of propanoic acid or trimethylamine, which altered the internal pH of cells. For the in vivo measurement, HEK293 or MIN6 cells were used. Then, AC8-fused Ci/Ce Epac2-camp was tested in MIN6 cells. The fluorescence along the cell membrane was observed. Depolarization induced by KCl treatment increased the cAMP level and, as a result, the R/R0 value increased right after the addition of KCl. Finally, HEK293 cells over-expressing voltage-gated Ca^2+^ channels (VGCCs) showed a large change in the R/R0 than the dummy control upon KCl treatment, implying AC8 activation.

Intracellular ions could change the FRET ratio, leading to quantification difficulties. The mCerulean/mCitrine FRET pair was fused to the Epac1-based cAMP sensor [48]. The existing Epac1-based cAMP sensor with ECFP/EYFP was greatly affected by H^+^ and Ca^2+^ ions, giving incorrect ratiometric FRET signals. The modified sensor was able to overcome this problem to give a consistent ratiometric FRET value over certain ion concentrations (H^+^, Ca^2+^, and Cl^−^) in live NIE-115 cells. This sensor was fast-matured (after 6 h of transfection) and long-lasting (more than 14 h) compared to the existing one which needed 10 h for maturing and only lasted 4 h. In addition, the dynamic range of the mCerulean/mCitrine FRET pair was 10% wider than that of eCFP/mCitrine. The aggregation problem in live cell imaging was greatly reduced by using monomeric citrine and monomeric cerulean. Finally, aggregation was not observed in primary hippocampal neurons for 4-day transfection with this sensor, while severe aggregation was observed with the existing one.

Fourth generation Epac-based cAMP sensors were developed based on the original one. The newly developed fluorescent protein mTurquoise2 [49] and mCerulean3 [50] were tested as donors and cpVenus, cpCitrine, citrine, tandem cpCitrine, and tandem cpVenus were tested as acceptors [51,52]. U2OS cells were transfected with these sensors and FRET spectral imaging was performed. The in vitro FRET assay was done using HEK293T cells. As a donor, mTurquoise2 was better than mCerulean3, regarding brightness. Tandem cpVenus was the best choice as an acceptor. Thus, the mTurquoise2-Epac-cpVenus-cpVenus construct was the best fourth generation FRET sensor. Dark acceptors were also tested for fluorescence lifetime imaging (FLIM). The construct with tandem dark cpVenus showed the best performance. Finally, mutation Q270E in Epac1 increased the cAMP affinity by about 2.4-fold in in vitro cell homogenates. The same sensor was used to generate transgenic mice under the CAG promoter [53]. AAV-Cre-virus-infected neurons revealed that the sensor was localized all over the cell, including in the nucleus, cell body, endosomes, and Golgi, among others. The application of forskolin and IBMX decreased the FRET signal. In contrast, the depletion of cAMP by cell membrane permeation led to an increase of the FRET signal. Treatment of striatal neurons with several neurotransmitters changed the FRET ratio, reflecting the cAMP dynamics. In addition, cAMP signaling in neurons could be modulated by adenosine. In combination with the optogenetic method, real-time cAMP dynamics of the striatal circuitry was investigated in brain slices using two-photon microscopy. This method can be used to study G-protein coupled receptors (GPCR) signaling pathways in real time.

Epac1, Epac2, and PKA RβII were fused directly to ECFP and EYFP, to generate several fusion proteins [54]. In purified protein experiments, Epac1-camp, Epac2-camp, and PKA-camp all underwent a decrease of the EYFP/ECFP fluorescence ratio in the presence of cAMP. CHO, COS-7, and HEK293 cells were transfected with these sensors to study the cAMP dynamics induced by its agonist. All three sensors have similar activation properties, but Epac1-camp had a better performance than the other two. In hippocampal neurons and peritoneal macrophages, cAMP signals induced by the β–adrenergic receptor moved quite fast (40 μm/s) across the cell.

A cAMP sensor using Epac 1 was generated to study the compartmentalization of cAMP signaling. ECFP (donor) and citrine (acceptor) were fused to the N-and C-terminals of Epac2 and Epac1 [55]. The best construct with the largest FRET signal change was named as ICUE1. Upon isoproterenol treatment, the FRET ratio decreased. The subcellular localization of the sensor was imaged using various targeting sequences, including CAAX (plasma membrane), NLS (nucleus), MitoDAKAP1 (mitochondria), and MitoCOX (mitochondria). Sensors were observed at the expected locations, proving the ability of monitoring cAMP dynamics in the subcellular compartments. Finally, it was shown that there was a delay between the immediate response of cAMP at the plasma membrane and the PKA response in the nucleus.

ICUE1 was modified to make an improved version of ICUE2 (the first 148 amino acids in Epac1 were removed), which improved sensitivity and exhibited minimal disruption in normal cell functions [56]. In the in vitro assays with lysed HEK293 cells, an EC_50_ = 12.5 μM was calculated and 61% of FRET ratio change was obtained. Upon treatment with isoproterenol and forskolin, the FRET ratio decreased in HEK293 cells, while the R373E mutant did not show such response. β_2_-AR receptor stimulation by isoproterenol induced a dose-dependent cAMP response; however, the rising–decaying kinetics were almost the same for all the concentrations tested. PDE inhibition by IBMX elongated the cAMP response time, meaning that PDE is important in regulating the cAMP level in cells. Furthermore, silencing or ablation of β-arrestin 1 or 2 reduced the β-AR inactivation, leading to a cAMP level elevation.

Membrane rafts are microdomains containing high cholesterol and sphingolipids that form unique regions in the cell membrane. Since they are related to the regulation of several cellular processes, especially in neurological and cardiovascular diseases, they have attracted much attention as a therapeutic target. Targeting sequences sometimes affects the dynamic range of the FRET sensors. For example, the FRET ratio decreased by ~50% when the CAAX sequence was tagged to ICUE2. Citrine in ICUE2 was replaced with cpVenus to overcome this drawback and named ICUE3 [57]. In HEK293 cell experiments, a 67% improvement in the dynamic range was achieved over ICUE2. ICUE3 was also membrane-localized via Lyn kinase. This sequence made sensors localized at the membrane rafts. Methyl-β-cyclodextrin (MβCD)-induced cholesterol depletion resulted in an increase of β_2_-AR-mediated cAMP accumulation in HEK293 cells. ICUE3 was also used to study PKA holoenzyme composition in real-time [58]. Regulatory subunits were conjugated to CFP (RII-CFP) and the catalytic subunit was conjugated to YFP (C-YFP). Isoproterenol changed the FRET signal only by a small degree for 400 s, representing a little dissociation of PKA holoenzyme. However, cells pre-incubated with rolipram before the β-AR agonist were shown to have a dramatic change in the FRET ratio. This result is strong evidence that PKA holoenzyme dissociates only if cAMP concentration reaches a certain level.

Epac2-camp was transfected into adult ventricular myocytes of Guinea pig to measure the basal cAMP level, which was found to be ~1.2 μM [59]. However, only the cytosolic cAMP level was counted to this value since cAMP from the compartments hardly affected the global value change. Thus, even though the global changes in the cAMP level can be induced by β_1_ or M_2_ receptor activation, the cAMP level in the microdomains was maintained much below that of the cytosol, by compartmentation.

Epac2-camps exhibited a fast photo-bleaching, which made it difficult to be applied to long-term live cell imaging due to the continuous decrease of the S/N ratio [60]. A newly developed sensor modified the GFP/mCherry FRET pair to enhance the photostability and increase the S/N ratio. The CFP/YFP FRET pair in Epac2-camps was substituted by the GFP/mCherry pair. Additionally, a K405E mutation in the cAMP-binding domain reduced the *K_d_* from 900 to 300 nM. *Xenopus laevis* natural tube cultures were transfected with both Epac2-camps and the new sensor. Even though the dynamic range of the new sensor was slightly narrower than that of Epac2-camps, the photostability was dramatically increased. After 1000 frames of imaging, only ~40% of the initial fluorescence was decreased in the case of GFP and mCherry, while in the case of CFP and YFP, it decreased ~60%.

Epac1-camp sensor was modified to study cAMP dynamics in the vicinity of cardiac ryanodine receptors type 2 (RyR2) [61]. The cytoplasmic N-terminus of junction (JNC) was fused to the sensor. Transgenic mice expressing this sensor in adult myocardium were imaged, and isolated adult ventricular myocytes were imaged upon the addition of isoproterenol. The CFP/YFP ratio increased after the addition of isoproterenol, representing an elevation of the local cAMP level. Next, isolated ventricular cardiomyocytes from both healthy and diseased transgenic mice were stimulated with β-adrenergic agonist isoproterenol and selective β_1_-or β_2_-adrenergic receptor blockers. The local cAMP level increased due to β_2_-adrenergic receptor stimulation in cardiomyocytes from diseased mice, but not in cardiomyocytes from healthy mice. In addition, the PDE2 inhibitor had no effect on the RyR2 microdomain, while the contribution of PDE4 was higher here than in the cytosol. In diseased cardiomyocytes, the effects of PDE2 and PDE3 in the microdomains increased, while that of PDE4 was significantly reduced.

An assay for cAMP binding to CNBD was performed using Epac1-CFP and 8-Fluo-cAMP, which is a fluorescein-conjugated cAMP [62]. Since the emission spectrum of 8-Fluo-cAMP largely overlaps with the excitation spectrum of CFP, this sensor could be used as a FRET sensor. Using this sensor, the binding of cAMP to CBND was studied including the binding with/without a competitor, evaluation of association/dissociation rate constants, and the competition curve with other cNMPs. *K_d_* of the sensor was 3.65 nM with/without imidazole, and FRET efficiency upon cAMP-CNBD binding was ~80%. This kind of binding-based sensor would be useful to investigate protein–ligand binding with the FRET technique.

There is a protocol for the calibration of the FRET ratio, by transforming the FRET ratio into an absolute cAMP concentration, by measuring the actual permeability of cAMP analogs, and by precisely determining the cAMP affinity of the sensors. CFP-Epac1-YFP sensor was used for this purpose [63].

For sensitized emission (SE) detection, CFP is a good donor; however, GFP would be a better donor for fluorescence lifetime imaging (FLIM) detection. A new FRET-based cAMP sensor, ^T^Epac^VV^, has mTurquoise2 as a donor and cpVenus-Venus as an acceptor [64]. HEK293 cells expressing this sensor showed cytosol-localized fluorescence signal. This sensor showed an optimal performance for both SE and FLIM experiments with an increased dynamic range and an enhanced photostability. For biological application, ^T^Epac^VV^ was expressed in HEK cells, then PGE_1_, isoproterenol, and IBMX+forkskin were applied sequentially. All three stimuli induced significant change in FRET ratio. NIE-114 and HeLa cells also showed similar results upon those stimuli.

The representative disadvantages of hyperspectral imaging were the low signal and the bad temporal resolution. An image mapping spectrometer (IMS) that does not require sample scanning and that enabled full spectral information from each pixel to be processed simultaneously was developed using “snapshot”. This device has a high collection efficiency (~65%) and a fast acquisition rate (7.2 fps max.). To test the device, the above mentioned ^T^Epac^VV^ cAMP sensor and GCaMP5G Ca^2+^ sensor were utilized [65]. Even though ECFP, EGFP, and EYFP have a significant spectral overlap, a linear unmixing algorithm was able to clearly re-make each spectral image. Then, the same sensors were transfected into MIN6 cells to image [Ca^2+^] and [cAMP] oscillation in real time. [Ca^2+^] and [cAMP] oscillations were recorded after 5 min of stimulation with glucose and triethanolamine (TEA). Each component of ^T^Epac^VV^ (mTurquoise2 and cpVenus-Venus) was able to be traced using an IMS. Furthermore, the correlation between [Ca^2+^] and [cAMP] oscillation was investigated.

#### 2.2.3. Other Sensors 

Hormones and neurotransmitters that bind to GPCR are important molecules responsible for biological signal transductions. They have different time scales in their response to stimuli. GPCR signaling dynamics were investigated in live cell using α_2A_-AR (binds to neurotransmitters) and parathyroid hormone receptor (PTHR, binds to PTH). Several insertion positions of the CFP/YFP were tested in the third intracellular loop and C-terminus of both receptors [66]. The best constructs were named as PTHR-cam and α_2A_AR-cam. PTH-cam (1 μM) induced the FRET ratio to decrease with a time constant τ = 3.00 in HEK293 cell experiments. The same result was obtained with noradrenaline. Kinetic analysis revealed that the activation is much faster in α2A-AR in an ms time scale than in PTHR.

Bacterial cyclic nucleotide-binding domain (mlCNBD) [67] was inserted between the N-terminal citrine and the C-terminal cerulean to create a CFP-YFP FRET-based cAMP sensor. This domain has an affinity of approximately nanomolar (~nM) for cAMP [68,69]. Using a purified mlCNBD-FRET sensor, 35~40% of the FRET value changed when cAMP with a higher concentration than 1.0 μM was added. However, a concentration higher than 2 μM of cGMP also induced a 20~25% change. The best FRET change occurred between a pH of 7.5~8.0, and the *K_d_* of mlCNBD-FRET for cAMP and cGMP was 66 and 504 nM, respectively. Response kinetics of the sensor was measured by using the stopped-flow method. The on and off rates of cAMP binding were 2.5 M^−1^s^−1^ and 9.3 s^−1^, respectively. HEK293 cells expressing this sensor were stimulated with a mixture of NKH477 and IBMX to study cAMP dynamics in mammalian cell lines. In addition, the reversibility of the sensor was tested by alternating the application of isoproterenol and a wash-out step. To investigate cAMP dynamics in sperm flagella, transgenic mice were generated [70]. The injection of bicarbonate induced an increase of the FRET ratio, which means it increased the free cAMP. cAMP in HEK293 cell cilia was monitored by expressing the sensor fused with somatostatin receptor 3 (SSTR3) domain. The treatment of isoproterenol induced a FRET ratio change, while the buffer-only control did not.

Even though many FRET-based cAMP sensors have been developed so far, a contradiction between the cAMP concentration in the cell lysate (1 μM) and the EC_50_ of PKA (100~300 nM) has not been solved fully. Five kinds of FRET-based cAMP sensors (EPAC-S^H187^, CUTie, AKAP79-CUTie, C9H6, and AKAR3) were tested in CHO cells to solve this discrepancy [45]. Previous in vitro measurements yielded lower values, compared to the intact cell measurement (at least one order of magnitude). The direct measurement of the cAMP levels and PKA activation in mammalian cell lines resulted in an estimated PKA activation constant which is 20-times larger than that obtained from the in vitro assay.

Two orthogonal FRET sensors were developed to do co-imaging of cAMP and PKA signaling dynamics in live cells [71]. For PKA signaling dynamics imaging, the A-kinase activity reporter (AKAR) was sandwiched between cerulean and mCherry fluorescent proteins (CR-AKAR). For cAMP signaling dynamics imaging, Epac1 was sandwiched between mCherry and mVenus (YR-ICUE). HEK293T cells expressing CP-AKAR underwent a ~1.4-fold change in the FRET ratio upon treatment with 50 μM forskolin and so did YR-ICUE. After stabilization of the signal, PKA inhibitor H89 was added and the CP-AKAR FRET ratio decreased, while the YR-ICUE signal was not affected.

Hyperpolarization-activated cyclic nucleotide-gated potassium channel 2 (HCN2) was fused to both CFP and YFP to monitor cAMP compartmentation in adult cardiomyocytes [72]. It has a high dynamic range and sensitivity, and is localized in the cytosol. The EC_50_ of this sensor for cAMP was found to be 5.9 μM from the HEK293A cell experiment. Isoproterenol treatment decreased the FRET ratio, which meant that it elevated the cAMP level in the cytosol, and the original value was recovered after removal of isoproterenol. Cardiomyocytes were isolated from transgenic mice. Cells emitted fluorescence in both the CFP and YFP channels, compared to wild-type cells. Using fluorescence recovery after the photobleaching (FRAP) technique, the diffusion coefficient of cAMP in cardiomyocytes was measured to be about 7 μm^2^/s. Finally, the difference in the cAMP level after stimulation by two β-adrenergic receptors (β_1_AR and β_2_AR) was observed in adult cardiomyocytes.

## 3. Single Luciferase-Based cAMP Sensors 

**Figure 2:**
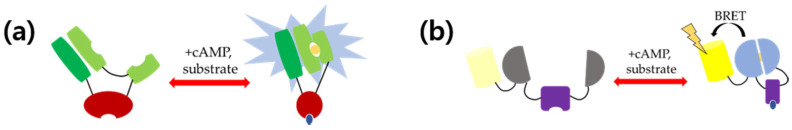
Schematic representation of structure and working mechanism of (**a**) single luciferase-based cAMP sensor 22F and (**b**) nano lantern-based cAMP sensor. Binding of cAMP initiates assemblage of firefly luciferase in 22F, leading to oxidation of substrate. In the nano-lantern-based cAMP sensor, binding of cAMP initiates assemblage of Renilla luciferase. Excitation by oxidation of substrate leads to energy transfer to Venus, resulting in yellow fluorescence emission.

The cAMP-binding domain from PKA (RIIβB) was inserted between the N-terminal fragment and the C-terminal fragment of circularly permuted *Photinus pyralis* (firefly) luciferase. An initial construct, 20F, was successfully applied to monitor the cytosolic cAMP. However, it underwent saturation under certain experimental conditions in live cell imaging, and the brightness decreased in cells other than HEK293 [73,74]. A total of 11 point mutations were done: Five in the N-terminal luciferase fragment and cAMP-binding domain and six in the N-terminal linker. The new sensor 22F had a larger dynamic range (~35-fold) and a broader range (0.003~100 μM) that showed a linear response than those of 20F [75]. Figure 2a shows conformational change of 22F upon binding of cAMP and in the presence of luciferin. In live-cell imaging with HEK293 cells, this sensor was able to trace the signaling dynamics in the presence of full, partial, or inverse agonists.

Several group II and group III metabolic glutamate receptor (mGluR) genes were stably transfected into CHO cells expressing the above sensor [76]. Modulation of the cAMP level by group II and group III mGluRs was observed. cAMP assay kits using luciferase, which do not require the consumption of ATP, are also commercially available [77,78,79].

Low-level laser treatments have been used for better wound healing, pain relief, and several inflammatory conditions [80,81,82,83,84,85]. Even though the exact and concrete mechanisms are not known, low-level laser irradiation (LLLI) is thought to be associated with mitochondria, in which the respiratory chain reaction occurs. Mitochondrial signaling is modulated through the membrane potential, which leads to the generation of reactive oxygen species (ROS), Ca^2+^ influx, and cAMP generation. Upon 830 nm laser illumination, the luminescence intensity did not change until 1200 s, which means that the cAMP level was elevated by LLLI [86]. In contrast, the luminescence intensity gradually decreased in the control groups. This effect was also observed when using a 658 and 785 nm laser, but not in the control. In addition, an 830 nm laser irradiation with adenosine receptor antagonists also elevated the cAMP level, compared to the control.

## 4. BRET-Based cAMP Sensors 

Even though FRET-based cAMP sensors have been shown to be remarkably effective, there are intrinsic limitations in these systems. First, one needs to use excitation light to obtain a fluorescence signal from the donor and acceptor, which is usually unfavorable for living species. Second, light of shorter wavelengths has to be used to excite donor FP (dark blue for CFP, blue for GFP and YFP), which might be toxic due to the higher energy per photon. In addition, the excitation light cannot penetrate deeply enough for animal imaging, such as in *Xenopus* or mice. To overcome the problems originating from using an excitation light, scientists have developed new systems using chemical luminescence. There are organisms emitting bioluminescence by oxidizing the substrate in the presence of ATP. Firefly, click beetle, Renilla, copepod, bacteria, and dinoflagellate are the representative species that have luciferase. Since luciferase emits luminescence by oxidizing the substrate chemically, no excitation light is needed, compared to FPs.

Renilla luciferase has a higher *k_cat_* (4.4) than that of firefly luciferase (0.1) [87]. However, its quantum yield is too small (0.053) [88]. As a result, emission from luciferases is too weak compared to the fluorescence, when expressed in living species. *Renilla reniformis*, a sea pansy also expressing its own GFP, overcomes low-emission problem by pairing luciferase with GFP [89]. Energy from the excited luciferase (donor) by oxidation of its substrate, coelenterazine, is transferred to the Renilla GFP (acceptor). This process is similar to FRET; thus, it is called bioluminescence resonance energy transfer (BRET). Since the quantum yield of Renilla GFP is 0.3, the emission efficiency of the BRET pair increases by 6-fold. New probes, including aequorin-GFP and BAF-Y, showed an enhanced performance, but they still suffer from an insufficient output photon number compared to FP-based probes [90,91].

### 4.1. Epac1-Based cAMP Sensor 

Trace amines (TA) are associated with a certain family of GPCR in the mammalian central nervous system. To study trace amine associated receptors (TAAR), however, one needs to solve the poor membrane expression problem and understand the molecular basis of ligand binding. The existing FRET sensor ICUE2 was modified to use luminescence [56]. Cyan fluorescent protein at the N-terminus was substituted by Renilla luciferase. Together with citrine at the C-terminus, the BRET signal was monitored in real time in HEK293 cells [92]. Upon the binding of cAMP, the luminescence decreased due to the conformational change of Epac1 and vice versa. More than 30 molecules were tested with this BRET-based cAMP sensor to determine the TAAR1 agonists. Nine out of 36 molecules were found to be agonists of TAAR1 (*p*-Tyramine, Octopamine, Tryptamine, *l*-Amphetamine, (+)-MDMA, *d*-Methamphetamine, Phentermine, 3-Methoxytyramine, and 4-Methoxytyramine). 

The same BRET sensor was used to study the D2 class dopamine receptors long isoform (D2_L_R)-mediated activation of G_i/o_, which inhibits cAMP in HEK cells [93]. In addition, the effect of antipsychotics on the β-arrestin 2-mediated signaling pathway was monitored. Bernard Masrl et al. found that antipsychotics used in their experiments had no activity in D2_L_R induced β-arrestin 2 recruitment. Indeed, most antipsychotics were inverse agonists at the G_i/o_ pathway. In the other experiments, TAAR1-mediated stimulation of cAMP by β-phenylethylamine (β-PEA), a kind of TA, was enhanced by D2R antagonists and these phenomena were recorded as the BRET changed [94]. The sensor also recorded that this effect on TAAR1 signaling was disrupted by pertussis toxin.

cp229Citrine was used to develop the cAMP sensor using YFP-Epac-RLuc (CAMYEL) [95]. It showed a 2-fold BRET ratio change compared to the one using the original citrine. The BRET ratio changed about 70% upon binding of cAMP. It has a *K_d_* of 8.8 μM, which is similar to that of the FRET sensor. Even though Ca^2+^ can affect luciferase activity, up to 1 mM of Ca^2+^ did not affect BRET ratio. Jiang et al. found that sphingosine 1-phosphate (S1P) indirectly regulates cAMP, by binding G_s_-coupled receptors. CAMYEL was also used to study human gonadotropin receptors, follicle-stimulating hormone receptor (hFSHR) and luteinizing hormone receptor (hLHR) [96]. When these two receptors were co-transfected with CAMYEL, BRET changes were observed when their ligand hFSH and hCG were bound to them.

### 4.2. Nano Lantern-Based cAMP Sensor 

Nano-lantern was developed by the fusion of mutant RLuc and YFP (Venus) [97]. Improved RLuc which has a mutation, and a truncated N-terminus was used along with Venus with truncated C-terminus. This fusion protein is much brighter and smaller than any other BRET pairs. Based on the nano-lantern, a bright cAMP indicator was developed by inserting the cAMP-binding domain Epac1 between the two hundred and twenty-eighth and two hundred and twenty-ninth residues of RLuc8. Figure 2b shows conformational change of the nano lantern-based cAMP sensor upon binding of cAMP and in the presence of its substrate. The purified protein showed a 130% signal change upon cAMP binding. *Dictyostelium discoideum* cell lines stably expressing the nano-lantern (cAMP) sensor (*K_d_* = 1.6 μM) were tested to monitor the intracellular cAMP concentration changes. The luminescence signal increased rapidly by 40%, and then gradually decayed, reflecting cytosolic cAMP dynamics. Spikes appeared periodically with 6-min intervals when stably expressing cells were imaged on a culture plate for 60 min. However, single *Dictyostelium discoideum* cell imaging failed due to the low expression level in this amoeba.

### 4.3. PKA-Based cAMP Sensor 

RLuc and GFP were used as a donor and acceptor for the PKA-based cAMP sensor [98]. Unlike other fusion protein cAMP sensors, a donor fusion protein and acceptor fusion protein were made individually and co-transfected for BRET imaging. The catalytic subunit of PKA (C-subunit) was conjugated with GFP and two different regulatory subunits (RI and RII) were conjugated with RLuc. COS-7 and HEK293T cells were co-transfected with an equal amount of the two constructs to monitor PKA holoenzyme formation in live cells. GFP-C subunit/RI-RLuc combination was the best PKA type I BRET pair. Likewise, the GFP-C subunit/RII-RLuc was the best one. Next, PKA subunit dynamics were tested with cAMP and several cAMP analogs. In wild-type PKA type I, 65~70% of the PKA sensor was dissociated, while more than 90% of the PKA type II sensor was dissociated upon forskolin/IBMX application. Both types of enzymes with mutations did not show a BRET change upon all the tested molecules. In the in vitro test with the COS-7 cell lysate, the PKA type I holoenzyme showed a full dissociation, which was in contrast with the in vivo results. However, the mutant holoenzyme did not show any response. Even though the RI-R210K mutant protein did not show a dissociation inside the cell, it was completely dissociated in vitro, despite the fact that the kinetics was slower than in the wild-type one. Table 1 is the summary of some promising cAMP sensors.

## 5. Conclusions and Prospects 

In summary, genetically-encoded cAMP sensors for non-invasive live cell/animal imaging were reviewed. The most popular sensors were the ones utilizing Epac as a cAMP-sensing domain. Sensors with a single FP or a luciferase were the simplest sensors with a minimal size and minimally perturbed the target proteins. FRET-based sensors were the most actively researched tools due to their good dynamic range and large pool of available FPs. However, the conversion of the FRET ratio into an accurate cAMP concentration sometimes needed calibration, which made it difficult to use. In addition, excitation light needed to be used to excite the donor and obtain a FRET signal. BRET biosensors, however, did not need an excitation light, since donors were naturally occurring or synthetic luciferases, whose luminescence relied on the chemical oxidation of substrates. However, they did need the injection of substrates such as luciferin. The selection of the best sensor required consideration of several aspects, such as the purpose of the experiments, the types of data needed, target species, target domains (or proteins), experimental conditions, and so on.

Almost all FP-based sensors had disadvantages in long-term imaging, since they underwent photo-bleaching. The development of more photo-stable FPs might elongate imaging time by reducing photo damage. FRET sensors usually use blue or green light for excitation. However, these short-wavelength lights had high energy per photon, which made them not suitable for long-term imaging due to their cytotoxicity. In addition, penetration depths of these lights were not long enough for deep tissue imaging or animal imaging. Thus, BRET-based sensors could be alternative methods, since they did not require an excitation light. So far, no BRET-based cAMP sensor using RFP has been developed, since the excitation spectra of RFP and the emission spectra of luciferase did not largely overlap. It is worth considering RFPs with a large Stokes shift to be excited by luciferase.

Finally, the simultaneous mapping of cAMP level fluctuation from several micro or nanodomains with a much-improved accuracy and stability would be challenging. Because cAMP levels are tightly regulated locally, and these compartmentalized cAMP dynamics induce a number of signal transduction at the same time, genetically-encoded probes that can be applied to investigate these dynamics will broaden our scope of the understanding of the cellular processes.

## Figures and Tables

**Table 1:** Summary of some promising cAMP sensors.

Method	Structure	Properties	*K_d_*	Application	References
Single FP	cpGFP-Epac2	Upward/downward	10~100 μM	Baculovirus, mammalian cell	[12,13]
Single FP	cpmNeonGreen-Epac2, cpmApple-Epac2	Green/Red	NA	Mammalian cell	[14]
Single FP	mApple-mEpac1	Fast response time, red fluorescence	7.2 μM	Mammalian cell, mice	[24]
Single FP	cpmApple-PKA	High affinity	0.3 μM	Mammalian cell, amoeba	[27]
FRET	CNBD(N)-YFP-CNBD(C)-CFP	Consistent dynamic range,	NA	Mammalian cell, cardiac myocyte, rat	[37,38,39]
FRET	cpVenus-PKA-ECFP	Large dynamic range	37.2 nM	Mammalian cell, amoeba	[40]
FRET	mTurquois2-Epac1-cpVenus-cpVenus	Bright, good affinity	9.5~4.0 μM	Mammalian cell, mice, FLIM	[51,52,53]
FRET	cpVenus-Epac1-Cerulean	Good dynamic range	NA	Mammalian cell	[58]
Single Luciferase	FLuc(N)-PKA-FLuc(C)	Large dynamic range, broad range of linear response	NA	Mammalian cell	[73,74,75]
BRET	RLuc-Epac1-Citrine	Real-time dynamic assessment	NA	Mammalian cell	[92]
BRET	cpCitrine-hEpac1-RLuc	Good dynamic range	8.8 μM	Mammalian cell	[95,96]
BRET	Venus-RLuc(N)-Epac1-RLuc(C)	Bright, small	1.6 μM	Amoeba	[97]

CNBD: Cyclic nucleotide-binding domain. FLuc: Firefly luciferase, RLuc: Renilla luciferase, hEpac1: Human Epac1.

## Data Availability

Data sharing not applicable.
